# Understanding Digital Learning Behaviors: Moderating Roles of Goal Setting Behavior and Social Pressure in Large-Scale Open Online Courses

**DOI:** 10.3389/fpsyg.2021.783610

**Published:** 2021-11-26

**Authors:** Peng Zhang

**Affiliations:** ^1^Xi’an University of Technology, Xi’an, China; ^2^Xi’an Technological University, Xi’an, China

**Keywords:** digital entrepreneurship, learning behavior, technological applications, goal setting behavior, sustainable digital economy

## Abstract

Pandemic has changed the whole scenario worldwide, not only related to business but also has equally affected the education sector. The classes have gone online from their physical nature, making it more convenient for students to learn. They provide online courses and lectures at the convenience of teachers and students. This study has also been one such effort in identifying the role of technological applications, intentions, and time flexibility in the digital learning behavior of students in China. The sample used in this study was the students taking online courses through their universities. The sample size was 343 students selected through purposive sampling. Smart PLS 3.3.3 has been used for data analysis *via* structural equation modeling. This study has found that technological applications play an important role in digital learning behavior, positively moderated by goal-setting behavior. Similarly, intentions predict digital learning behavior. Moreover, social pressure has also been found to augment the role of time flexibility in digital learning behavior. These results are very useful for universities that make understanding the online nature of studies more comprehensive.

## Introduction

As a result of the spread of coronavirus disease 2019 (COVID-19), countries worldwide have taken unprecedented actions in various areas to combat the pandemic. This scenario also impacted education, resulting in the largest disruption of educational systems in history. Currently, most countries have announced extended closings, prohibiting almost 1.6 billion children and teenagers from attending school. However, several European Union organizations and international organizations have long advocated for digital technologies in education. The COVID-19 influenced closure has an impact on education and learning, as well as instructional methods. Nevertheless, e-learning quickly filled this void, as schools, universities, and academic facilities shifted their learning and teaching to the internet. Due to its particular benefits, digital learning in China is advancing quickly, and its long-term viability has become increasingly important.

While digital learning has been quickly developing in recent years, China is experiencing a digital learning surge due to the pandemic. Because people spend greater time at home than before, digital learning has become an essential educational resource. The pandemic has shifted the traditional chalk and board teaching paradigm to one based on digital technology. The number of people taking online courses has increased dramatically, and this trend is certain to continue. Regulators are currently attempting to increase involvement at a massive scale while guaranteeing that digital Learning solutions are accessible to all. However, it would be naive to believe that digital learning is progressing smoothly. Regarding the relationship between students and teachers, there is much that can be done to improve virtual coaching platforms and online education, just as there is in physical classrooms. So far, digital learning has raised some queries and suggested some future directions of online learning.

Digital education, as one of the most important components of quality education, has unique qualities that support the long-term development of education, such as flexibility, low cost, repetition, ease, low threshold, high efficiency, broadly accessible users, and rich instruction. These advantages give digital learning an advantage over conventional classroom learning (Isaac et al., 2019; Khalil et al., 2021). Similarly, digital learning can open up new learning opportunities for new students while significantly altering learning provision and the competitive environment. Digital learning overcomes time and location restrictions by offering educational opportunities to distant learners and allowing flexible learning modes, helping students to freely select and pace their learning paths by their actual circumstances, as well as an advantage from contingent teaching (Moore et al., 2011). From the distance education model just at the turn of the 21st century to the present popular internet education model, the concept and pattern of digital education have changed considerably. It has also evolved beyond the distribution of fixed content to the usage of dynamic, public classrooms, and digital training (de Souza Rodrigues et al., 2021). There are some technological applications of digital learning. The benefits of learning paired with educational technology include accommodating slow learners in more individual ways and the ability to promote the learning enthusiasm of students in undertaking exercises or projects assigned by teachers. The use of digital technology in education is thought to improve results and inspire students individually, based on the benefits achieved. Improving digital learning outcomes is a direct indicator of the effectiveness and efficiency with which learning is implemented. As a result, the development of learning through the use of technological advancements is critical, and the teacher or educator should use it as much as feasible. It is necessary to avoid turning this psychological development into a negative experience, reflecting poorly on educators and pupils. The way of human development is employed will determine whether it has positive or negative consequences (Kirkwood and Price, 2006). Students will rapidly become bored if the display of the learning content is not effectively designed or looks like a learning textbook.

Furthermore, due to their poor knowledge of technology, teachers who do not comprehend the application of technology will be unable to create learning using this technology. The role of the teacher is limited to that of a facilitator, whereas pupils must improve their capacity to comprehend the information or topic offered by the teacher. For students to learn joyfully and passionately. Collaboration can be aided by educational technology (Sarfraz et al., 2018; Shehzad et al., 2020). Teachers can interact with students throughout class, but students can also speak with one another. Students collaborate to solve challenges through online lessons and learning games. Students can share their views and ideas and encourage one another in collaborative tasks. At the same time, technology allows students to communicate with teachers physically. Students can ask questions about what they’re learning in class and get extra help with subjects they don’t grasp. Students can upload their homework from home, and teachers can use their laptops to access and view completed assignments.

Despite the rapid advancement of Web technology in teaching, student enthusiasm for using technology in the classroom is waning. With the tremendous technological advancements in China, the Internet is becoming more significant in many sectors of life, including education. Despite the flexibility, ease, and ingenuity of the Internet when contrasted to conventional teaching methods (Chen, 2018). Despite enormous expenditures in new technology by governments, universities, and service providers, the full advantage and value of digital learning platforms have yet to be realized (Barclay et al., 2018). This involves deploying ongoing research into the factors that influence student happiness (Herrador-Alcaide et al., 2019). On one side, universities, authorities, and service providers might use authentic and reliable methodologies to target areas that need to be modified or improved based on the determinants of student happiness, thereby improving the quality of online learning services (Cidral et al., 2018).

Educators, course creators, and training developers, on the other hand, can profit from such studies to give learners the necessary online learning environments and more appropriate online learning programs. Instead of the other way around, online students can organize their study time around the rest of their day. You can work when convenient for you, allowing you to balance employment and family obligations while continuing your education. Students have complete control and accountability over their learning when they use flexible learning. Rather than being forced to attend a class, people can choose when and how much time they spend learning. This power allows them to plan their education around their obligations and assures that they are studying at the optimal moment. For instance, some learners may be more productive in the evenings, but conventional education may limit them by only providing classes throughout the day.

Flexible learning allows students to determine how and when they will learn by customizing their course to their specific needs. They also benefit from learning at their own pace, which can assist in relieving a lot of stress. Teachers frequently rush through subjects before giving pupils an assignment to complete. This might put pressure on individuals to finish tasks fast, but it leaves no room for learners to ask questions. Suppose a student does not understand a concept or idea that the teacher has communicated. In that case, they will not accomplish the assignment to their full potential, obstructing their learning. Because of the flexibility of online learning, people can study their time grasping subjects and ensuring complete knowledge before moving on. Students are involved in reviewing their assessment findings, working with their teachers to develop reasonable but ambitious objectives for growth, and attempting to drive their education with regular reference to those goals, which is just one of many types of student-involved data use.

These goal-setting strategies positively impact student results and school cultures when they are properly applied. It is natural for students to be impacted by their peers as they navigate new social structures such as friendships, dogmas, and where they fit in when they first enter university. Peer pressure may influence students to do or say things they would not usually do or say. It is not always a terrible thing: societal influence to study more or take a stand against harassment can result in great outcomes (Sarfraz et al., 2020, 2021). On the other hand, some factors can be harmful, such as societal pressure to treat others badly or engage in risky behavior like binge drinking. Social conditioning can affect the self-esteem of a student and cause them to feel isolated from their friends and family. Some practical ways learners can be assisted include promoting a culture of diversity and inclusion, fostering open dialogs with students and parents about peer pressure, developing critical communication skills to help manage negative peer pressure situations, and building resilience. All these factors have a significant impact on digital learning. Hence, to understand digital learning behaviors, this study was designed and executed.

This study revolved around certain objectives as follows: (1) to estimate the digital learning behaviors among students; (2) to evaluate the impact of technological applications on digital learning of the students; (3) to analyze the role of flexibility in timings on digital learning behaviors of students; (4) to identify and check the significance of moderating factors such as goal setting and social pressures toward digital learning.

## Literature Review

### Impact of Technological Applications on Digital Learning

In digital learning, technology has been widely used to assist instructors in accomplishing various educational goals, and adaptable technologies can help them achieve their aims (Kreijns et al., 2013). Digital technology has the advantage of being very scalable. This is also true in education, where huge classrooms are still a preferred mode of instruction due to their cost effectiveness around the globe (Vermeulen et al., 2017). Due to various potential cost efficiency and adaptability, digital technologies have acquired a lot of traction in education. While several new digital technologies have been studied for adoption, there are very few comparable similarities (Sarfraz et al., 2018; Budd et al., 2020). Technological quality control, which can predict behavioral intention to utilize technology, is the most well-established approach for measuring adoption. The student response method received the most positive feedback. E-lectures were next, supported by classroom discussion, and finally, a portable virtual reality (Kisin, 2021). CRSs, also characterized as student evaluation systems, personalized response systems, immediate reply systems, digital response systems, clickers, even public response systems, have such a wide range of applications in the classroom. A CRS lets lecturers ask mobile telecommunication questions earlier, throughout, and then after their presentations, so students can respond using their own electronic devices (Sprenger and Schwaninger, 2021). The responses are compiled in real-time and displayed to individual students or the entire class. This helps lecturers to keep track of the comprehension of their students when it comes to topics they discuss.

Furthermore, the mental capacity of students is typically 20 min (Muir et al., 2020). Students can chat with each other because lecturers speak in front of the class. The front channel and covert operation are two types of communication that happen at the same time. Students who seem timid and shy have benefited from using computer tools that allow them to remain anonymous, particularly when the themes are problematic (Asarta and Schmidt, 2020). Several universities have adopted the practice of documenting lectures by using different technological applications.

Students were provided access recordings that enabled them to examine topics through their own time and create a learning environment (Lasfeto, 2020). Digital literacy, portable voice, and online chatting are just some of the multimedia instructional networks that have sprung up due to the rapid expansion of the World wide web and modern communications technology (Tohara, 2021). Traditional education would be replaced by using the accessibility and attractiveness of the technology to use digital teaching resources to achieve national competitiveness. As a result, a great deal of research into mobile learning is being done to provide better system performance and widespread use. Keeping in view the literature, the following hypothesis was formulated.

H1: Technological applications have a positive impact on digital learning behavior.

### Impact of Online Learning Intention on Digital Learning

From student intention and capacity perspectives, views or beliefs, and online learning situations, studies have been undertaken on the continuing intention of university students when it comes to learning online (Zulfiqar et al., 2021). Furthermore, few studies have examined how the factors will combine and influence the intentions of students to learn digitally (Chang et al., 2017; Sarfraz et al., 2020). The researchers looked at how the view of individuals on online learning changed over time and the connections between their personality learning abilities, interaction with other users, perspectives, and online learning intention (Zhu et al., 2020). University online courses, given in either a digital or mixed modality, have seen a substantial increase in enrollment in recent decades as online learning better supports the different demands of students by trying to break down based on geographical obstacles (Herodotou et al., 2020).

Academic institutions, in particular, have seen a shift in an image from completely digital training to differentiated instruction, which is now a well-established component of university education (Li et al., 2020). Given the rapid expansion of blended education in higher education, one major difficulty has suddenly appeared: preserving the intention and good views of students toward online learning. Researchers used specific models that focus on the attitudinal characteristics of the participants, significantly and positively associated, learning intention, or online course outcomes in previous studies on the continuing online learning intention of university students (Syauqi et al., 2020). The study was carried out in an online learning environment.

The technology acceptance model (TAM) is a paradigm for determining the desire of a student to be using technology and participate in online learning (Husain et al., 2020). The perceptions of learners when it comes to utility and simplicity may also influence their desire to keep their education online. The expectation confirmation model (ECM) was based on the ECT (expectation confirmation theory), technology acceptance model (TAM), and theory of planned behavior (TPB) Expectations of customers and perceptions of product performance, according to the ECT, may play a role in post-purchase satisfaction (Avotra et al., 2021a,b). Many studies looked into learning motivation theories and discovered that learning intention elements affected the desire of students to learn online. The ECT and fairness theory discovered that interactional justice, interpersonal communication fairness, achievement value, perceived utility, and inherent value all influenced the intention of students to learn online (Cicha et al., 2021). The effectiveness of positively predicting digital learning behavior is measured using online learning intents as a major benchmark. Online learning intention is related to digital learning behaviors so, the following hypothesis was developed.

H2: Online learning intentions positively predict digital learning behavior.

### Impact of Flexible Timing on Digital Learning

Students can work at their own pace with digital learning, and there are chances to encourage active instructional practices (Scully et al., 2021). Digital Blended learning necessitates strong self-regulated learning (SRL) abilities due to the large online component since learners must interact with internet resources and study independently (Broadbent et al., 2021). Educational strategies have been developed depending on the use of tactics or how the other learner employs specific tactics (Tohara, 2021). Digital learning entails weekly repetitions of online and face-to-face elements throughout a program. Learners are given digital materials to gain basic analysis of the changing topical module at their speed through the active internet connection. The face-to-face component entails instructional strategies and higher-order reasoning guided by an instructor, allowing students to practice and apply what they learned during the online preparation.

As the dissemination of data among two specific parties takes precedence, among the most important features of e-services is the availability of information (Shkarlet et al., 2020). Most academics believe that it is a widespread perception of the capability of the internet that it is mostly utilized to fulfill the passion for learning and the need for knowledge in the educational sector (Sein-Echaluce et al., 2020). E-learning satisfaction is the most important aspect of the e-learning of a student, supported by e-learning instructor quality, course materials selection, and e-learning administrative and service supporting quality (Mousavi et al., 2020). Technologies, especially information technology in the context of e, have changed the face of education in the knowledge economy (Oke and Fernandes, 2020). Institutions of higher learning face both challenges and opportunities as a result of the latently coming move away from the traditional model of teaching and training. One of the defining characteristics is the advent of communication and information technology (ICT), which has changed the nature of education like other industries. The following hypothesis was structured to check the significance of the impact between flexible timing and digital learning.

H3: Flexible timings have an impact on digital learning behavior.

### The Moderating Role of Goal Setting

Digital technologies benefit those who utilize them and, as a result, influence their behavior. Technology is transforming the way professors teach, and students learn. Management and faculty understanding of learning technology (LT) is influenced by technology, which changes the type and degree of adoption employed during education (Syed et al., 2021). With the advent of digital learning, digital literacy (DL), and digital communication literacy, the structure of information distribution transformed. Digital learning is a type of information delivery that employs technology to teach educational purposes (Sayaf et al., 2021). This type of technology makes use of web-enabled gadgets and represents digital learning (Aditya, 2021). While DL is defined as the capacity to use technology to locate, evaluate, generate, and convey information, it necessitates academic, behavioral, and technical expertise (Taylor et al., 2021). Disruptive innovation occurs when a particular technologies attempt to replace established and standard processes, resulting in unanticipated effects (Huo et al., 2021). University leaders require digitally literate employees to manage the frequent and rapid changes in technology that support administration and instruction. The scant research available examines the level of digital learning among rural community college employees (Hromalik et al., 2021). Although technology allows for quick responses and direct feedback from students, digital learning adoption and comprehension go beyond academic achievement. Keeping in view the literature, a hypothesis was developed on the moderating role of goal setting which is as follows.

H4: Goal setting behavior moderates the relationship of technological applications and digital learning behavior.

### The Moderating Role of Social Pressure

Many are engaging in technology-assisted complementary work, aided by numerous collaboration platforms that allow communication from any location or time (TASW). The challenges of balancing work and non-work time have been exacerbated by a global epidemic that has disrupted typical work schedules and locales (Goldman, 2021). Because of the COVID-19 epidemic, employees worldwide witnessed a sudden change in their work positions. For many workers, this meant redefining the distinction between works and associated with introducing (García-Peñalvo, 2021). Nowadays, ICTs provide workers with additional connectivity with individuals, teams, and organizations, which is a particularly important and influential feature (Richey et al., 2005). Workers can engage with one another without time limits or the necessity for co-location by using mobile phones, desktop computers, and communications connectivity.

Employees are increasingly working outside of traditional working hours, at night, or on weekends, thanks to the adaptability and accessibility of information and communication technologies (Albano et al., 2021). Collaboration technologies are distinct from other organizational technology in that they enable workers and organizations to be connected at all times. The capacity to monitor the activities of others contributed to advancements in who workers asked for task advice, according to the use of a common IT system by computer specialists. Employees will have additional opportunities to engage in supplemental work if these techniques are used frequently and intensely, as no technology hurdles are blocking these practices (Chittenden, 2021). Collaboration solutions, such as Google Workspace or Microsoft 365, comprise a variety of apps that allow remote coworkers to share files, update information individually or collaboratively, and communicate synchronously *via* video and conference calls. Typically, these technologies are used to facilitate collaboration. The moderating role of societal pressures was analyzed under the following hypothesis.

H_5_: Social Pressure moderates the relationship of flexible timings and digital learning behavior.

**FIGURE 1:**
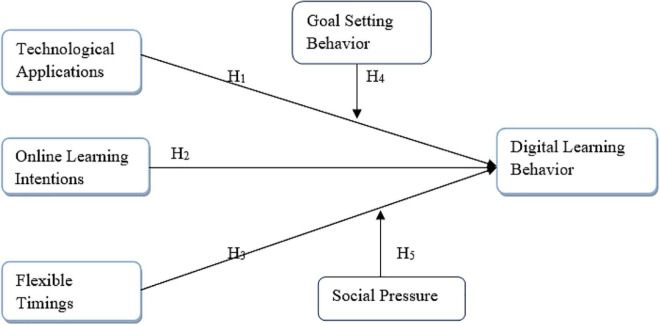
Conceptual model.

Based on these hypotheses, the following conceptual framework was designed (Please see Figure 1).

## Methodology

### Sampling and Instrument Development

Standard error of the mean (SEM) has been used in this study for data analysis to achieve the objectives. The data was collected through the technique of questionnaire. Data collection for this cross-sectional study took place in the universities of China. The respondents filled these questionnaires with their free consent. The population used in this study was the students at universities taking online courses during the pandemic. The sample size *N* for this study was 343, which were selected through purposive sampling. Since the pandemic was in the whole country, so all students were going through online learning. This sample is considered enough (Liébana-Presa et al., 2020), considering the general guidelines.

The data obtained during data collection was segregated based on frequency and percentages regarding the categorization of each question in demography. The results can be seen in Table 1. In respondents, there were 188 men and 155 women. Under the question of age, 139 respondents were under 25 years, while 46 were between 25 and 30, 79 respondents between 31 and 40, 56 between 41 and 50, and 23 over 50 years. Similarly, for the education question, 32 respondents were from higher secondary education, 115 from bachelors, 128 from masters, and 68 belonged to doctorate and other categories (Please see Table 1).

**TABLE 1:** Demographic summary.

Demographic summary	Frequency	Percentage
**Gender**		
Male	188	54.81
Female	155	45.18
**Age**		
<*25*	139	40.52
25–30	46	13.41
31–40	79	23.03
41–50	56	16.32
50>	23	6.70
**Education**		
Higher secondary	32	9.32
Bachelor	115	33.52
Masters	128	37.31
Doctorate	66	19.24
Others	2	0.58
**Fields of study**		
Management	111	32.36
Social Sciences	137	39.94
Natural Sciences	95	27.69

*N = 343.*

Questionnaires used for data collection were consist of 32 items in total, representing six variables. The flexible timings variable consisted of four items, digital learning behavior of eight items, goal-setting behavior of six items, online learning intention of six items, the social pressure of four items, and technological application variable consisted of four items. It was designed on seven points Likert scale with 1 being strongly disagreed, and 7 = strongly agree. The scale was adapted according to past research (Devries et al., 2018). This study contained three independent variables (technological applications, online learning intentions, and flexible timings), two moderators (goal-setting behavior and social pressure), and one dependent variable (digital learning behavior) (Lin and Chen, 2017; Devries et al., 2018). Data collection was done through online questionnaires depending upon the accessibility to the internet and availability. To maintain the anonymity of the respondents, the data obtained was saved on the server having HTTP security. Through Smart PLS 3.3.3 (SmartPLS GmbH^1^), PLS-SEM was used to check the hypotheses.

## Data Analysis

The data collected were checked for the reliability and validity of the questionnaire. For reliability, two types of reliability were used, i.e., Cronbach alpha reliability and composite reliability. The alpha reliability of the variables ranged from 0.849 to 0.93, which meets the threshold of 0.7. Similarly, the composite reliability is also from 0.883 to 0.943. These results can be seen in Table 2.

**TABLE 2:** Measurement model and descriptive statistics.

Constructs	Code	FD	α	CR	AVE
Flexible timings			0.921	0.943	0.806
	FT1	0.870			
	FT2	0.913			
	FT3	0.880			
	FT4	0.927			
Digital learning behavior			0.930	0.942	0.671
	EB1	0.851			
	EB2	0.827			
	EB3	0.827			
	EB4	0.825			
	EB5	0.829			
	EB6	0.783			
	EB7	0.801			
	EB8	0.808			
Goal setting behavior			0.871	0.883	0.559
	GS1	0.811			
	GS2	0.663			
	GS3	0.713			
	GS4	0.636			
	GS5	0.844			
	GS6	0.795			
Online learning intention			0.849	0.833	0.470
	OLI1	0.502			
	OLI2	0.538			
	OLI3	0.502			
	OLI4	0.622			
	OLI5	0.936			
	OLI6	0.875			
Social pressure			0.921	0.944	0.808
	EB1	0.919			
	EB2	0.901			
	EB3	0.867			
	EB4	0.908			
Technological apps			0.896	0.927	0.762
	TECH1	0.883			
	TECH2	0.861			
	TECH3	0.873			
	TECH4	0.874			

*FD, Factor Loadings; CR, Composite Reliability; AVE, Average Variance Extracted; α, Cronbach Alpha reliability; FT, Flexible Timings; DLB, Digital Learning Behavior; GSB, Goal Setting Behavior; OLI, Online Learning Intention; SP, Social Pressure.*

Moreover, for the validity of data, factor loading for each variable was also obtained as defined criteria (Nawaz et al., 2019; An et al., 2021). The factor loadings for all variables were above 0.8 except for the online learning intentions variable that showed as low as 0.502, which is also acceptable in certain cases (Khalil et al., 2021). These results for factor loadings can also be seen in Table 2.

**FIGURE 2:**
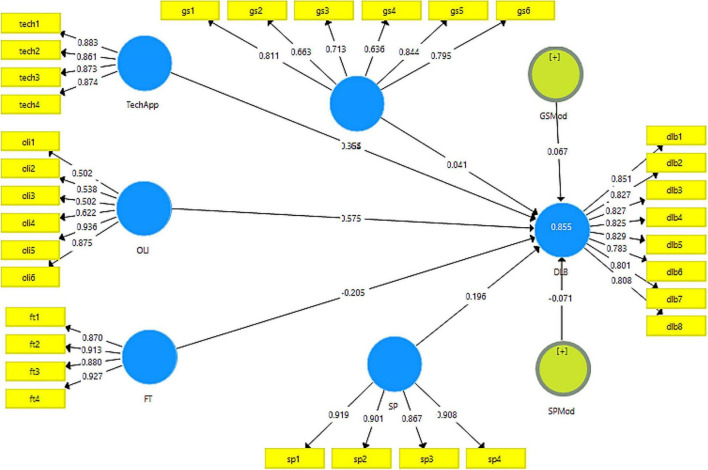
PLS-Algorithm for measurement model.

The factor loadings obtained to check the validity can be seen in Figure 2, obtained through PLS-Algorithm for the measurement model.

The data was further validated through the Fornell and Larcker criterion of correlation, as shown in Table 3. To validate data, each top value given in each column should be higher than the rest of the values underneath (Cepeda-Carrion et al., 2019). The topmost value for DLB is 0.819, FT is 0.898, GSmod is 0.71, OLI is 0.686, SPMod is 0.782, and TechApp is 0.873, hence, meeting the said criteria for validity. Similarly, another measure to validate the data is through the HTMT ratio. This test was also run on the data and the results can be seen in Table 4. The values for heterotrait-monotrait ratio should be less than 0.9 for data to be valid (Cepeda-Carrion et al., 2019). The values in this study are less than 0.9 hence, the data is valid. The highest ratio for HTMT was found at 0.887, which is between the DLB variable and TechApp variable. The rest of the rations are even lesser than this (see Table 4).

**TABLE 3:** Fornell and larcker criterion.

Variables	DLB	FT	GSMod	OLI	SPMod	TechApp
DLB	**0.819**					
FT	0.268	**0.898**				
GSMod	–0.386	–0.276	**0.710**			
OLI	0.804	0.580	–0.425	**0.686**		
SPMod	–0.177	–0.211	0.395	–0.161	**0.782**	
TechApp	0.812	0.272	–0.536	0.721	–0.200	**0**.**873**

*FT, Flexible Timings; DLB, Digital Learning Behavior; GSMod, Goal Setting Behavior as moderator; OLI, Online Learning Intention; SPMod, Social Pressure as moderator; TechApp, Technological Application.*

**TABLE 4:** HTMT ratio.

	DLB	FT	GSMod	OLI	SPMod	TechApp
DLB						
FT	0.278					
GSMod	0.325	0.286				
OLI	0.627	0.876	0.424			
SPMod	0.177	0.210	0.447	0.158		
TechApp	0.887	0.288	0.490	0.604	0.207	

*FT, Flexible Timings; DLB, Digital Learning Behavior; GSMod, Goal Setting Behavior as moderator; OLI, Online Learning Intention; SPMod, Social Pressure as moderator; TechApp, Technological Application.*

**FIGURE 3:**
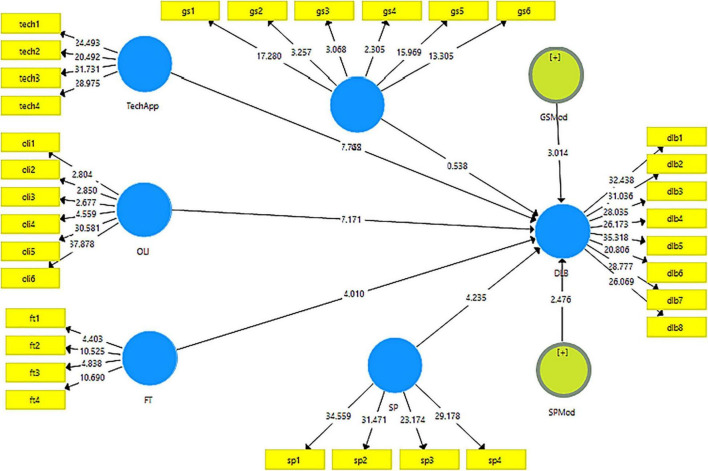
*Consistent* PLS-bootstrapping.

Furthermore, the data was checked for the direct effects of the variables through a structural model using PLS consistent bootstrapping method (Lemes et al., 2021; see Figure 3). Interestingly, *t*-statistics for all the hypotheses were found significant. The detail is presented in Table 5.

**TABLE 5:** Results for structural model.

Paths	H	T-Stats	*P*-Value	Adjusted R^2^	Results
TechApp → DLB	H1	7.742	0.000***	0.852	Supported
OLI → DLB	H2	7.171	0.000***		Supported
FT → DLB	H3	4.010	0.000***		Supported
GSBMod → DLB	H4	3.014	0.003***		Supported
SPMod → DLB	H5	2.476	0.014**		Supported

*Significance level*

***0.005%,

**0.05%, H, Hypothesis; O, Original Sample; M, Sample Mean; SD, Standard Deviation; E&T, Education and Training; ESE, Entrepreneurial Self-efficacy; IM, Intrinsic Motivation; EB, Entrepreneurial Behavior.

All the results obtained were found significant at *p* < 0.005 except for H_5_ which was significant at *p* < 0.05. For the first hypothesis, technological applications were found to have the most significant effect on digital learning behavior with *t*-statistic 7.742 followed by online learning intention with t-statistic 7.171. Flexible timings have also been found to play an important role in predicting digital learning behavior (*t*-statistic = 4.01). Moreover, goal-setting behavior was found to trigger the role of technological applications in predicting digital learning behavior (*t*-statistic = 3.014). The moderation of social pressure in predicting digital learning behavior has also been significant in enhancing the role of flexible timings. These results can be seen in the following table. Overall, all these independent variables predicted digital learning behavior at 85.2%, indicating the vitality of these variables for DLB (Please see Table 5).

## Discussion

This research was based on several hypotheses to analyze digital learning behaviors with moderating roles of goal-setting behavior and social pressure in large-scale open online courses. Similarly, this study has also been one such effort in identifying the role of technological applications, intentions, and time flexibility in the digital learning behavior of students in China. Among two major approaches for conducting the research, structural equation modeling was carried out using Smart PLS. A theoretical framework was designed, and questionnaires were sent to the participants. The results supported the hypotheses. The results were also in accordance with many researchers, and some were of a different opinion. The possible reasoning for the obtained results is also discussed here. 55% of the respondents were men and 45% were women. They all had different education levels ranging from higher secondary to Doctorate from management sciences, social sciences, and natural sciences.

The cut-off value for reliability is said to be 0.7 (Haq and Awan, 2020). All the values in this study are above 0.7 ranging from 0.849 to 0.93 for alpha reliability and 0.883 to 0.943 for composite reliability. Hence the data in this study is reliable. The maximum threshold stated in the literature for factor loadings is 0.6 (Howell et al., 2018; Linkov et al., 2018), All the values in this study are above 0.8 except the online learning intentions variable that showed as low as 0.502 which is also acceptable in certain cases. The possible reason for getting these results was the authenticity and reliability of the data collected from the participants. Discriminant validity was also tested and found satisfactory for the research. This is also due to the authenticity of the data. For the other criterion i.e., HTMT ratio, the researchers agree that the value should not exceed 0.9, i.e., all values should be less (Cepeda-Carrion et al., 2019). The results for this study meet this criterion hence, making the data valid for use. In the third phase of data analysis, the data were analyzed for structural model or path analysis using bootstrapping with Smart PLS 3.3.3.

This is usually the subsequent stage of the measurement model. The significance of the relationships is usually expressed in the form of path analysis, which either shows the direct effects or the indirect effects. The direct effects are the general linear regression, however, indirect effects indicate the mediating variables. For the first hypothesis, technological applications were found to have the most significant effect on digital learning behavior with a *t*-statistic 7.742. This is because technological applications are the most important contributors to digital learning. Many past researchers had shown similar results in their findings (Zhu et al., 2020; Zulfiqar et al., 2021). The second highly significant result was obtained in the hypothesis of online learning intention with *t*-statistic 7.171. This is due to the fact that intention plays an important role in learning through any medium. The results are also in favor of many researchers such as (Scully et al., 2021). Flexible timings have also been found to play an important role in predicting digital learning behavior (*t*-statistic = 4.01). The most amazing feature of digital learning is the flexibility in the timings of learning due to which, this hypothesis may have been significant toward digital learning of the students. Moreover, goal-setting behavior was found to trigger the role of technological applications in predicting digital learning behavior (*t*-statistic = 3.014). This is also due to the fact that goal-setting behaviors are the root cause of many successes. These results are also in accordance with many researchers such as (Alabdulaziz, 2021). The moderation of social pressure in predicting digital learning behavior has also been significant in enhancing the role of flexible timings. Overall, all these independent variables 85.2% predicted digital learning behavior, indicating the vitality of these variables for digital learning behaviors. All hypotheses were supported in this study. This happened due to the fact that all these factors influence the digital learning behaviors of the students.

## Conclusion

With the increasing use of the internet, the introduction of different technological applications and pandemics has changed the overall realm of learning and education in China and worldwide. This study is also such exploration into the learning behaviors of students considering the online nature of education. Students are also more prone to online learning showing vivid intentions for digital learning behavior. Furthermore, this has also freed the students from the strict timings of university, providing online recordings of the lectures available anytime. Moreover, goal-setting behavior and social pressures have augmented the digital learning behaviors among students. These findings have been an important milestone in this pandemic for teachers and institutes in making their course outlines and learning more effective for students. This research has several implications for future researchers and e-commerce players who are interested in repeating this research with their available resources in different regions. These can be exploited well in finding new avenues for certain research like this.

## Study Limitations

There are certain limitations of the study toward digital learning behaviors such as solo acts. In digital learning, students have to learn independently; they need personal coaching and contact with the instructor. No matter how difficultly we strive to convert verbal interactions to digital sites completely, and no matter how normal it appears to make connections across digital screens, an online reality can never be fully human. None can ever be a substitute for personal contact. Thirdly, constantly being connected to digital resources is the new normal. Still, the fact is that excessive usage of a laptop or tablet can lead to impaired vision, physical difficulties, and strain injuries. Fourthly, it is doubtful that your digital learning audience will be inspired to self-study if they have so little self-discipline. Lastly, there is always a possible lack of control so, there is no guarantee that your messages will be received, no matter how carefully you plan your eLearning course. You give your students autonomy over their digital learning experience, which is fantastic, but will they use it efficiently? There is still the possibility that students will just skim through the information without paying attention. These are some limitations that need to be kept in mind while designing further digital learning behaviors.

## Data Availability Statement

The original contributions presented in the study are included in the article/supplementary material, further inquiries can be directed to the corresponding author/s.

## Ethics Statement

All subjects gave their informed consent for inclusion before they participated in the study. The study was conducted in accordance with the Declaration of Helsinki, and the protocol was approved by the Xian Technological University, China.

## Author Contributions

PZ conceived and designed the concept, literature review, data collection and wrote the manuscript. The author has read and agreed to the published version of the manuscript.

## Conflict of Interest

The author declares that the research was conducted in the absence of any commercial or financial relationships that could be construed as a potential conflict of interest.

## Publisher’s Note

All claims expressed in this article are solely those of the authors and do not necessarily represent those of their affiliated organizations, or those of the publisher, the editors and the reviewers. Any product that may be evaluated in this article, or claim that may be made by its manufacturer, is not guaranteed or endorsed by the publisher.

## Footnotes

1
https://www.smartpls.com
